# A Radiographic Evaluation of Missing of Permanent First Molars in a Group of Iranian Children and Adults: A Retrospective Study

**DOI:** 10.1155/2018/5253965

**Published:** 2018-04-01

**Authors:** Mostafa Rezaie, Jannan Ghapanchi, Abdolaziz Haghnegahdar, Leila Khojastehpour, Hooman Khorshidi, Heshmatollah Heidari

**Affiliations:** ^1^Department of Oral and Maxillofacial Medicine, School of Dentistry, Shiraz University of Medical Sciences, Shiraz, Iran; ^2^Department of Oral and Maxillofacial Radiology, School of Dentistry, Shiraz University of Medical Sciences, Shiraz, Iran; ^3^Department of Periodontics, School of Dentistry, Shiraz University of Medical Sciences, Shiraz, Iran; ^4^Department of Oral Health and Community Dentistry, School of Dentistry, Shiraz University of Medical Sciences, Shiraz, Iran

## Abstract

The missing of permanent first molars influences the occlusal status and dental health. The purpose of this study was to determine the prevalence of missing first molar teeth in a selected population of Shiraz, Iran. *Methods*. A total of 2206 panoramic views of patients aged from 7 to 75 years old were inspected for missing of permanent first molars. Patients were categorized into five age groups: from 7 to 15, 16 to 30, 31 to 45, 46 to 60, and more than 60 years old. Data were categorized according to sex, age, and number of lost teeth using SPSS software. *Results*. No first molar was missing in 59.9% of the cases, 17.05% had lost one, 10.4% had lost two, 7.2% had lost three, and 5% were missing all four of their permanent first molars. The mandibular first molar was the most commonly lost tooth, and the left side in both jaws was more affected than the right side. There was a positive relation between age and missing first molar. *Conclusions*. A missing first molar is a common finding in southern Iran population. Due to the important role of permanent first molars in occlusion, more education and dental care is recommended to preserve these teeth.

## 1. Introduction

Dental caries is a multifactorial disease that is influenced by several characteristics, such as diet, microorganisms, teeth morphology, and saliva. It is also influenced by social, environmental, and cultural factors. Epidemiological studies have shown that the prevalence and experience of dental caries have decreased in many regions of all age groups over the last three decades; however, this decline has not been the same in all countries [1–3].

A cariogenic diet and access to dental care may be highly correlated with the appearance of dental caries. Age, sex, education and dental health habits, and access to professional care can lead to a difference in the incidence of caries and periodontal disease in different social groups [1, 4]. Caries and periodontitis are mentioned as the main predictors of tooth missing [5–7]. The caries prevalence may be decreased even after reduction of fluoride concentrations in the water in some populations [8].

The permanent first molar teeth usually erupt when a child is six years of age. The first permanent molar is considered a key of occlusion, and its early loss due to caries can have a significant impact on the future dental health [9]. Early orthodontic and/or prosthetic treatment of the edentulous space is necessary to prevent or reduce the negative consequences of early extraction.

Al-Khateeb et al. [10] in Saudi Arabia studied the prevalence of caries and treatment needs among school children aged 6–12 years and concluded that the mean caries experience in deciduous teeth was 2.9 in private schools and 6.3 in public schools.

The present study used orthopantomographic images to determine the prevalence of first molar missing. The panoramic view is a screening method that has numerous diagnostic and treatment applications [11].

The aim of this study was to determine the prevalence of first molar missing in a selected population.

## 2. Materials and Methods

The present study was carried out by the Department of Oral Medicine and Radiology of Shiraz Dentistry School to assess the prevalence of the first permanent molars missing among patients aged 7–75 years. We used the files of these patients, but we had no history of the causes of the tooth loss. In this retrospective study, 2206 panoramic views of patients referred to a private clinic of maxillofacial radiology, between 2012 and 2014, were retrieved and reevaluated for missing first molars in a SCANORA station. Panoramic radiographs were acquired using a panoramic CRANEX SOREDEX digital orthopantomography device (Finland) set at 57–85 kVp and 10 mA.

All panoramic views of acceptable quality were entered into the study. The radiographs with severe overlapping or any other cause which resulted in unreliable diagnosis were excluded. All radiographs were classified according to the patient's age, sex, and the presence or absence of permanent first molars missing. In cases with missing teeth, all radiographs were categorized in order to show the quadrant and number of missing teeth (one quadrant missing a first molar and two quadrants, three quadrants, and finally four quadrants missing first molars). Patients were also categorized into five age groups: from 7 to 15, 16 to 30, 31 to 45, 46 to 60, and more than 60 years old, to provide a better evaluation (Table 1). In some cases, delayed eruption was seen, so we selected images from patients aged 7 years and more.

Since radiographs had been prepared for other dental purposes, no extra dose was imposed on the patients, and no names or personal information of the patients was recorded in the reports and figures, so no ethical limitations prevented the performance of this study.

After coding of variables (sex, age, and missing first molar), all of data were gathered and computerized. The SPSS software (SPSS Hong Kong) was used to describe the frequency and percentage of all patients. The chi-square, Mann–Whitney *U*, Kruskal–Wallis, and Spearman tests were used for data analysis for detection of statistical differences between groups according to age and sex. In all statistical analyses, *p* < 0.05 was considered statistically significant.

## 3. Results

A total of 2206 panoramic radiographs from 1507 female and 699 male aged between 7 and 75 years were examined. The age and sex distribution of the study participants are shown in Table 1.

A total of 59.9% radiographs in the overall sample did not show any missing teeth, 17.4% of the patients had one missing first permanent molar and 10.3% had two, 7.1% had three, and 4.9% had four missing first molars.

Missing mandibular first molars were seen in 741 (33.5%) of the subjects. Of these, 451 (20.4%) patients had one and 290 (13.1%) had two missing mandibular first molars. The right mandibular first molar was missing in 421 (19.1%) patients, and the left mandibular first molar was missing in 612 (27.7%). A maxillary first molar was missing in 500 (22.6%) subjects: 171 (7.8%) of them had one and 329 subjects (14.9%) had two missing maxillary first molars. In this group, missing right molars accounted for 397 (18%) and missing left molars for 426 (19.3%) of the panoramic views (Tables 2 and 3).

The chi-square test results showed a statistical difference and an association between gender and the prevalence of missing of permanent first molars (*p*=0.05). The Mann–Whitney *U* test showed a significant difference between the two sexes, with a male predilection for missing first molars (*p*=0.05); the mean rank for females was 1008.68, and the mean rank for males was 1130.79.

The Spearman test revealed a significant statistical difference and an association between age groups and the prevalence of missing of permanent first molars (*p*=0.05; correlation coefficient: 0.334). The Kruskal–Wallis test showed significant differences between the age groups (*p*=0.05). A direct and positive relationship was observed between the age groups and permanent first molars missing.

All groups were also compared to each other in order to determine the relationship between the age group and the number of tooth missing. A significant correlation was seen between the age groups and first molar missing number (*p*=0.05).

## 4. Discussion

In the present retrospective study, we investigated the prevalence of first permanent molars missing among various age groups and found that about 40% of the population studied had at least one missing of first molars. An increased prevalence of dental caries and periodontal disease may contribute to a high number of missing teeth in individuals [1, 12]. Access to dental care varies greatly among different social classes in Iran. The development of better treatment intervention strategies to reduce the incidence of dental problems—and consequently, to reduce the number of tooth extractions—depends on the development of epidemiological profiles of the population.

The present study focused exclusively on the first permanent molars since they play a key role in maintaining the dental and overall health of an individual. The first permanent molar is the first permanent tooth to erupt, and it exhibits a great control over the teeth that erupt later, behind and in front of it, as the other teeth are forced to position themselves with respect to the already erupted first molar, often leading to occlusion. The first molar is also the largest tooth in the oral cavity and bears the maximum occlusal load, and they influence the vertical distance of the maxilla and mandible, the occlusal height, and aesthetic proportions [9]. Since they have the maximum root surface area, they are also considered the best source of anchorage for moving the teeth. Above all, the health of these teeth, in particular, can form a good basis to assess the oral health status of a population [13].

Khazaei and others reported a high prevalence of tooth loss in Iranian adult population. They stated that tooth loss was more prevalent among men than women [14]. Jafarian and Etebarian in surveying the reasons for teeth extraction in Tehran, Iran, found that the dental caries and periodontal disease are the main reasons for tooth extraction and reported that the prevalence of extraction was high in men than in women [15]. In our study, 38.1% of women and 44.4% of men had at least one missing of permanent first molars.

The important finding in this research was that the lower left mandibular first permanent molar exhibited a statistically significant higher rate of extraction than the other first molars. This finding is in agreement with the studies of Farooqi and others in Saudi Arabia [16], who showed that the mandibular molars had a statistically significant higher DMFT than their maxillary counterparts. Note that, however, our study focused only on first molars missing and not on decay or filling rates. Another difference between the previous studies and our study was the wide age range in the current study and the limited age ranges in previous studies.

It seems that the morphology and the time of the eruption of mandibular first permanent molar lead to the higher rate of caries. The first molars of the mandible have many cavities and grooves that can act as food retentive areas for decay [17]. On the other hand, mandibular molars erupt slightly earlier than their maxilla counterparts, therefore exposing them to the oral cavity for longer times and causing them to be more prone to dental caries.

Most of the previous research [18–22] focused on patients less than 18 years of age and not this age group. Oliver et al. [22] reported that early loss of the first permanent molars, before the age of 15-16 years, was associated with a higher caries experience in adjacent teeth, particularly on the occlusal surfaces, which could lead to additional tooth extractions. The previous studies also reported that the loss of the first molars was associated with increased caries in the occlusal surfaces of adjacent teeth, which again could lead to more tooth extractions [22, 23]. These studies also reported that the missing of the first molars was associated with missing of the second molar and the second premolar. Our study evaluated only the rate of first permanent molars missing, so other changes in other teeth should be evaluated in future work.

Extraction of permanent first molars with a poor prognosis may be indicated during the mixed dentition in children [19, 20]. The extraction of permanent first molar(s) with subsequent orthodontic treatment in young patients has been considered an alternative to placing complex restorations [20]. In our study, about 24% of children from 7 to 15 have one missing molar, 31% have 2 missing molars, and 15% have three missing molars. The high prevalence of missing in the age group of 7–15 in the population under our study may be partly due to increased demand for orthodontic treatment [24] and the need for orthopantomographic images.

## 5. Conclusions

Missing of first molar is a common finding in this selected population in Shiraz, Iran. The different prevalence rates in the underage group (7 to 15 years old) compared with over-15-year-old groups is alarming that warrants attention by the government and policymakers. Due to the important role of this tooth in occlusion, more education and dental care is recommended to preserve these strategic teeth. This cross-sectional retrospective research is not an exact indicator of first molar missing, and further retrospective and prospective studies on larger populations and limited age ranges are needed to provide an accurate estimate of the number of missing first molars in this southern Iran population.

## Figures and Tables

**Table 1:** Frequency of first molar missing according to sex and age group.

	One missing	Two missing	Three missing	Four missing	No missing	Total
*Sex*						
Female	277 (18.3%)	131 (8.6%)	89 (5.9%)	77 (5.1%)	933 (61.9%)	1507 (100%)
Male	109 (15.5%)	97 (13.8%)	70 (10%)	34 (4.8%)	389 (55.6%)	699 (100%)
Total	386 (17.4%)	228 (10.3%)	159 (7.1%)	111 (4.9%)	1322 (59.9%)	2206 (100%)
*Age*						
7–15	29 (23.9%)	38 (31.4%)	18 (14.8%)	1 (0.8%)	35 (28.9%)	121 (100%)
16–30	108 (20.8%)	13 (2.5%)	0 (0%)	0 (0%)	398 (76.6%)	519 (100%)
31–45	115 (19.1%)	84 (13.9%)	29 (4.8%)	2 (0.3%)	371 (61.7%)	601 (100%)
46–60	102 (13.8%)	50 (6.7%)	72 (9.7%)	34 (4.6%)	479 (64.9%)	737 (100%)
>60	8 (3.4%)	32 (14%)	74 (32.4%)	74 (32.4%)	40 (17.5%)	228 (100%)

**Table 2:** Frequency of first molar missing according to jaw.

	Maxillary	Mandibular
No missing	1706 (77.3%)	1465 (66.4%)
One missing	171 (7.8%)	451 (20.4%)
Two missing	329 (14.9%)	290 (13.1%)

**Table 3:** First molar missing in four quadrants.

	Right maxillary	Left maxillary	Right mandibular	Left mandibular
Missing	397 (18%)	426 (19.3%)	421 (19.1%)	612 (27.7%)
